# 

**Published:** 1865-01

**Authors:** 


### INTERESTING ANALYSES OF ARTICLES IN FOREIGN JOURNALS.

### *The Relief of Near-Sight without Spectacles.* By J. V. SOLOMON, Esq., F. R. C. S.

ORIGINAL COMMUNICATIONS.......................................... 1
1. Tetanus and Tetanic Spasms, by M. H. Houston, P. A. C. S. 2. "Local Injuries Justifying the Amputation of a Limb," by Prof. Skey, St. Bartholomew's, abridged for publication by Frank A. Ramsay, Surgeon and Medical Director P. A. C. S. 3. Report of a Case of Dislocation upon the Dorsum Ilii, reduced after Twenty-five Days, by Manipulation, by A. M. Fauntleroy, Surgeon in Charge General Hospital, Staunton, Va. 4. Conservative Treatment of Compound Comminuted Fracture of the Femur, with Cases, by G. M. B. Maughs, Surgeon P. A. C. S. 5. On the Treatment of Camp Itch, by Assistant Surgeon S. R. Chambers.
EDITORIAL AND MISCELLANEOUS.................................. 11
Foreign Honors to an Army Surgeon. To Ascertain the Purity of Chloroform. London Punch on Army Surgeons—From an Army Swell. Hippophagy Again.
CHRONICLE OF MEDICAL SCIENCE.................................. 13
Address in Surgery, delivered at the Annual Meeting of the British Medical Association, by J. Paget, F. R. S. Clinical Lectures on a Case of Diabetes, delivered at St. Mary's Hospital, by Thomas K. Chambers, M. D., Lecturer on Clinical and Systematic Medicine at, and Physician to the Hospital. On the Treatment of Whooping-Cough by Belladonna and Sulphate of Zinc, by E. Garraway, Esq., M. R. C. S. E. On the Therapeutic Properties of Carbolic Acid, by C. Calvert, F. R. S. Alcohol from Coal gas.
INTERESTING ANALYSES OF ARTICLES IN FOREIGN JOURNALS.................................. 24
The Relief of Near-Sight without Spectacles, by J. V. Solomon, Esq., F. R. C. S. Origin of the Cow Pox. Galactagogues.

